# Suppression of Th17-polarized airway inflammation by rapamycin

**DOI:** 10.1038/s41598-017-15750-6

**Published:** 2017-11-10

**Authors:** Oana Joean, Anja Hueber, Felix Feller, Adan Chari Jirmo, Matthias Lochner, Anna-Maria Dittrich, Melanie Albrecht

**Affiliations:** 1 Department for Pediatric Pneumology, Allergology and Neonatology, Medical School Hannover, Carl-Neuberg-Str. 1, Hannover, Germany; 2 German Center for Lunge Research, BREATH Carl-Neuberg-Str. 1, Hannover, Germany; 3 Institute of Infection Immunology, TWINCORE, Centre for Experimental and Clinical Infection Research, Hannover, Germany; 4 Present Address: Department of Internal Medicine B, University Medicine Greifswald, Ferdinand-Sauerbruch-Str., Greifswald, Germany

## Abstract

Because Th17-polarized airway inflammation correlates with poor control in bronchial asthma and is a feature of numerous other difficult-to-treat inflammatory lung diseases, new therapeutic approaches for this type of airway inflammation are necessary. We assessed different licensed anti-inflammatory agents with known or expected efficacy against Th17-polarization in mouse models of Th17-dependent airway inflammation. Upon intravenous transfer of *in vitro* derived Th17 cells and intranasal challenge with the corresponding antigen, we established acute and chronic murine models of Th17-polarised airway inflammation. Consecutively, we assessed the efficacy of methylprednisolone, roflumilast, azithromycin, AM80 and rapamycin against acute or chronic Th17-dependent airway inflammation. Quantifiers for Th17-associated inflammation comprised: bronchoalveolar lavage (BAL) differential cell counts, allergen-specific cytokine and immunoglobulin secretion, as well as flow cytometric phenotyping of pulmonary inflammatory cells. Only rapamycin proved effective against acute Th17-dependent airway inflammation, accompanied by increased plasmacytoid dendritic cells (pDCs) and reduced neutrophils as well as reduced CXCL-1 levels in BAL. Chronic Th17-dependent airway inflammation was unaltered by rapamycin treatment. None of the other agents showed efficacy in our models. Our results demonstrate that Th17-dependent airway inflammation is difficult to treat with known agents. However, we identify rapamycin as an agent with inhibitory potential against acute Th17-polarized airway inflammation.

## Introduction

Different inflammatory diseases are associated with an increased Th17 response. In asthma, Th17-polarized airway inflammation correlates with severe, steroid-resistant airway inflammation and poor disease control^1^. In corresponding mouse models of allergic airway disease, Th17-associated airway inflammation was steroid-resistant as well^2^. Th17-polarized airway inflammation has also been implicated in cystic fibrosis, obliterative bronchitis, sarcoidosis and COPD, suggesting that several different pulmonary disease entities would benefit from a Th17-specific treatment approach^3^.

Interleukin (IL)-17A is a cytokine that exerts pleiotropic functions in the airways: it regulates the expression of pro-inflammatory mediators, particularly neutrophil-recruiting chemokines, mucus, bicarbonate, anti-microbial peptides and matrix metalloproteases (MMPs). Moreover, non-immune cells such as airway smooth muscle cells and fibroblasts are also responsive to IL-17A (summarized in^4^).

The effects of IL-17A in the respiratory tract are dual-faced: while IL-17A is necessary for successful defense against respiratory pathogens like *Staphylococcus aureus*, *Pseudomonas aeruginosa* and *Candida albicans*, it can also prompt severe lung damage and destruction, thereby contributing to lung function decline and, in some disease entities, respiratory failure (summarized in^4^).

This dichotomy turns suppression of a Th17-polarized airway inflammation into a double-edged sword: desirable in the attempt to treat steroid-resistant (allergic) airway inflammation but possibly deleterious by reduction of host defense mechanisms against a manifold of pathogens. Whilst anti-IL-17A antibodies have been licensed for clinical use against psoriasis, their effect in treating IL-17A dependent airway inflammation has been equivocal^5^ and might be fraught with significant side effects due to the loss of efficient pathogen control in the airways. Other IL-17A-specific anti-inflammatory agents are yet to be described.

The aim of this study was to assess different agents with known or possible efficacy against Th17-polarized airway inflammation. Most of these agents have been licensed or tested in clinical trials in different pulmonary diseases. To evaluate the anti-Th17 effects, we used murine models in which airway inflammation depends on polarized Th17 cells^6^. These models allow analysis of both acute and chronic airway inflammation, the latter being induced by priming of endogenous Th17 cells. Thus, these models provide instruments to dissect the effects of an agent on polarized Th17 cells versus *de novo* Th priming and an ensuing Th17 polarization.

We have chosen candidate substances for which published data suggested a Th17-inhibitory potential. Azithromycin, as a widely used immunomodulator in different pulmonary inflammatory diseases, has been shown to decrease airway neutrophilia and IL-8 secretion via inhibition of the IL-17A-IL-8 axis^7,8^. Similarly, we chose to evaluate roflumilast whose efficacy against neutrophilic inflammation has led to licensing for COPD and whose effects upon neutrophilia similarly rely on effects on the IL-17A-IL-8 axis^9^. Vitamin-A-agonism directly reduces Th17 differentiation by modulation of Foxp3 and ROR-gamma-t^10^ and was efficient in the treatment of other Th17-polarized diseases such as autoimmune encephalitis and type I diabetes^11,12^. We thus chose AM80, a retinoic-acid receptor/Vitamin A agonist, licensed for subtypes of leukaemia to evaluate its effects on Th17-dependent airway inflammation in our model. Finally, rapamycin, licensed for use against solid organ and bone marrow rejection, was shown to reduce Th17/Th1 polarization, leaving Th2 development largely unaffected^13^, and to reduce airway inflammation in conjunction with AM80^14^. Throughout our studies, methylprednisolone served as a negative control as published data^2^ and preliminary data in our model systems had revealed steroid-resistance of Th17-polarized airway inflammation.

## Materials and Methods

### Mice

BALB/cJ (WT) mice were purchased from The Jackson Laboratory. TCR-transgenic DO11.10 mice (C.Cg-Tg(DO11.10)10Dlo/J) backcrossed onto an αß−/− background and RORgt-reporter mice (Tg(Rorc-EGFP)1Ebe) were bred in our facility. Six- to 10-wk-old female mice were used in all experiments. All experimental methods described in this manuscript were performed as approved by the respective Institutional Animal Care and Use Committee (*Landesamt für Verbraucherschutz und Lebensmittelsicherheit*; protocols 13/1206 and 10/0297) and according to all relevant guidelines and regulations.

### Generation of polarized Th cells, adoptive transfer and ***in vitro*** restimulation

CD4+ T and syngeneic T-depleted splenocytes were prepared as decribed previously^15^. For generation of Th17 cells CD4+ T cells and APCs were cultured with 5 µg/ml pOVA323–339, 20 ng/ml recombinant murine IL-23 (eBioscience), 2 ng/ml recombinant human TGF-β (Peprotech), 40 ng/ml recombinant murine IL-6 (Miltenyi Biotec), anti-IL-4 (11B11) and anti-IFN-γ (XMG1.2). Cells were split 1:2 on day 3 and harvested on day 7. 5 × 10^6^ Th17 cells were injected i.v. into BALB/cJ mice. Purity before injection ranged from 92% to 98% CD4+KJ1-26+ and an additional aliquot of the cells was retained for *in vitro* restimulation and analysis by ELISA.

### Animal treatment protocol

**Figure 1:**
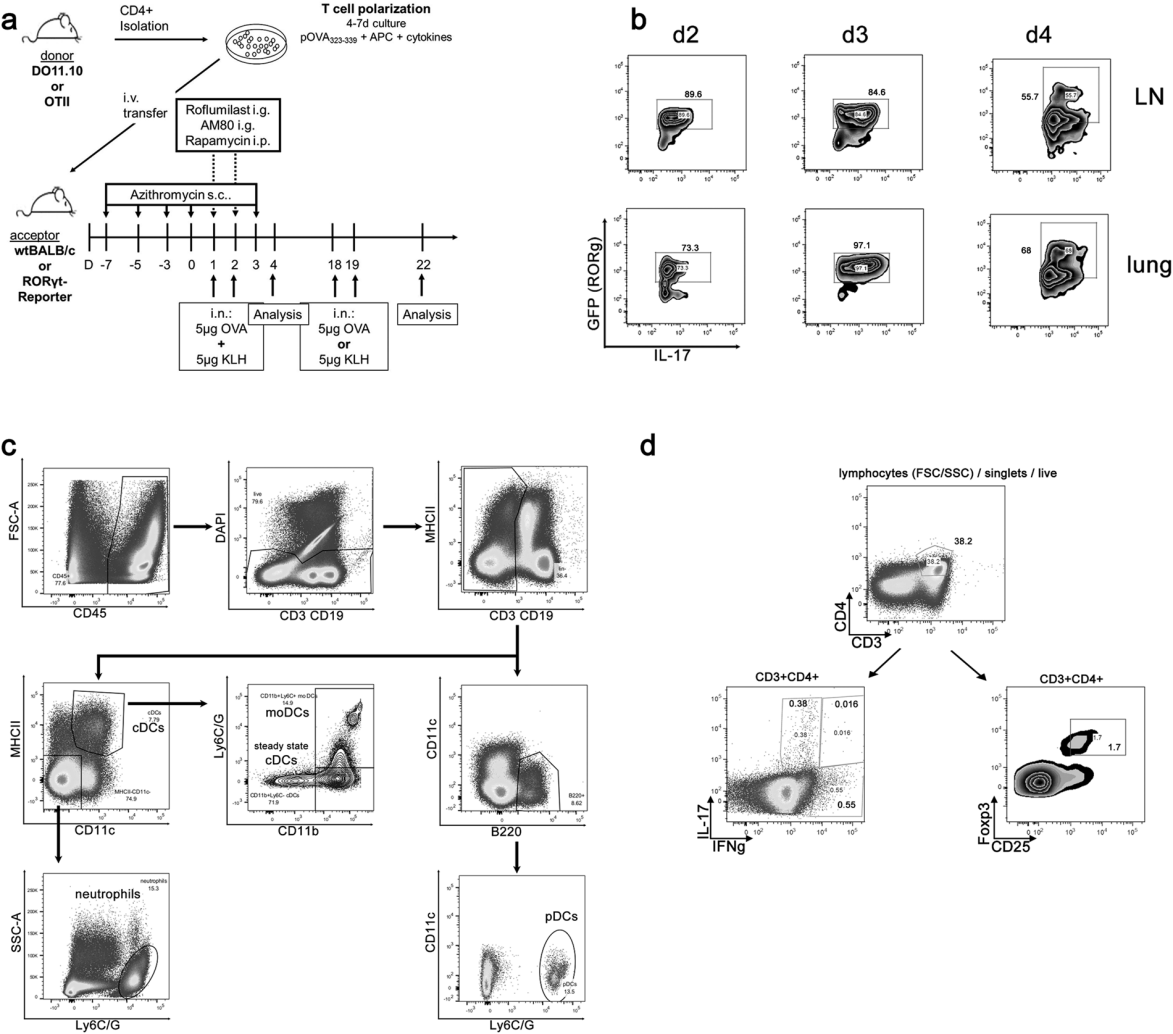
Model for Th17-dependent airway inflammation and flow cytometry gating strategies. (**a**) Mice receive polarized Th17-cells on d0 i.v. and antigen challenges are performed with OVA and KLH on d1, 2.Within the acute model, sacrifice takes place on d4. For the chronic model further challenges with OVA or KLH are performed on d18 and d19 and sacrifice takes places on d21.(**b**) Transfer of Th17 cells into ROR-gamma-t reporter mice demonstrates priming and polarization of endogenous IL-17A-positive lymphocytes in lung and lymph nodes. LN: lymph nodes. (**c**) Example of gating strategy: After gating out debris and gating on single cells we focused on live, (DAPI^−^, lineage negative (CD3^−^CD19^−^) leukocytic populations (CD45^+^). pDCs (CD11c^low^B220^+^Ly6C/G^+^ cells), cDCs (MHCII^high^CD11c^high^ cells) subdivided into steady-state cDCs (Ly6C/G^−^) and moDCs (Ly6C/G ^+^) and neutrophils (lin^−^MHCII^−^ CD11c^−^Ly6C/G^+^) were identified by appropriate surface markers. d: gating strategy T reg and cytokine production: After gating out debris and gating on single cells we focused on live (Pacific Orange negative) lymphocytes (FSC, SSC) T helper cells (CD3^+^CD4^+^). These were either analyzed regarding their IL-17A and IFNγ production or for the percentage of Tregs (CD25^+^Foxp3^+^).

For induction of *acute* Th17-dependent airway inflammation mice were exposed to 5 µg of OVA at days 1 and 2, 24 h after the transfer of Th17 cells. Mice were sacrificed on day 4 (Fig. 1a).

For induction of *chronic* Th17-dependent airway inflammation and assessment of treatment effects on priming of endogenous Th17 cells, mice were exposed to 5 µg of OVA (grade V; Sigma-Aldrich and 5 µg of KLH (Sigma-Aldrich) intranasally on days 1 and 2, 24 h after the transfer of Th17 cells. Secondary challenge was performed with either 5 µg of KLH or 5 µg OVA on days 18 and 19. Mice were sacrificed on day 22 (compare Fig. 1a). Performing the above protocol in RORγt-reporter mice^16^ leads to induction of ROR-γt positive T cells in LNs and lungs as early as d2 after transfer, demonstrating priming of endogenous Th17 cells within the recipients of polarized Th17 cells (Fig. 1b).

All pharmacological agents were titrated to effect with published references cited above serving as guidelines for titration doses. Time points of treatment were d1 and d2 of the treatment protocol for all agents except for azithromycin where a more prolonged treatment course (see below) was applied to resemble clinical application schemes more closely (Fig. 1a).

Roflumilast was applied intragastrically (i.g.) at a dose of 5 mg/kg in PBS^17,18^. AM80 was applied i.g. at a dose of 4 mg/kg in 0.5% CMC^12,19^. Rapamycin was applied intraperitoneally (i.p.) at a dose of 8 mg/kg in 0.2%PEG400/0.05% Tween^13,20^. Azithromycin was applied subcutaneously (s.c.) at a dose of 50 mg/kg in PBS on d-7, d-5, d-3, d0 and d3^21^. Methylprednisolone was applied i.p. at a dose of 25 mg/kg in PBS^22^. Appropriate vehicle controls were included in all experiments (Fig. 1a).

For induction of Th2-polarized airway inflammation, a standard house dust mite model (HDM) was used. Briefly, mice were sensitized by application of 100 µg HDM intranasally (i.n.) on d0 and challenged with 10 µg HDM i.n, on days 7–10. Inhibitors were applied as stated above 1 h before each challenge. Animals were analysed on d14.

### Analysis of BAL

BAL inflammatory cells were obtained by lavage of the airway lumen with PBS, prepared, stained and differentiated microscopically as previously described^23^.

### Determination of serum Ab concentration

Antigen specific (KLH, OVA) antibodies in sera were determined by ELISA as described previously^23^.

### Lymph node (LN) and lung cell preparation and restimulation

Mediastinal LNs and lungs were harvested on day 4 or 22 of collateral priming protocol (Fig. 1a) and pooled from each group at time of sacrifice. Single cell suspensions were obtained as described previously and cells were stimulated 200 µg/ml OVA, KLH or Medium for 72 h or 120 h^23^.

### Measurement of cytokine production

Cytokines and chemokine production was measured using either ELISA (R&D Systems), or cytometric beads array (Flowcytomix^TM^ (eBioscience)) as stated in the figures legends according to manufacturer’s recommendations.

### Flow cytometry analysis

All staining procedures were performed on ice. Cell surfaces were blocked with anti-FcR (24G2) antibody, followed by respective surface staining with monoclonal antibodies (see Supplementary Table 1 for complete list of antibodies) 0.33 µM 4′,6-diamidino-2-phenylindole (DAPI; Merck) or 1.3 µM Pacific Orange (molecular probes) was added immediately before analysis to discriminate live cells followed by filtering through 50 µm diameter CellTrics®(BD) disposable filters to ensure high quality FACS recording. Up to 5 × 10^6^ events were analyzed on a FACS Canto flow cytometer (405 nm, 488 nm, and 633 nm excitation) in association with FlowJo (Treestar) software.

For gating strategy refer to Fig. 1c,d. Briefly, analysis gates were set on live, single cells defined by scatter characteristics and exclusion of DAPI^+^ or PacificOrange^+^ cells. For DC analysis, we also excluded epithelial (CD45^−^) and T and B cells (CD3^+^ and CD19^+^) from our analysis. Dendritic cells were identified out of the non-autofluorescent CD19^−^CD3^−^ cell population and phenotyping was consecutively performed as follows: plasmacytoid dendritic cells were identified by B220^+^CD11c^low^Ly6C/G^+^ staining, conventional dendritic cells were defined as CD11c^high^MHCII^high^ cells which were further sub-divided in steady-state cDCs (Ly6C/G^−^) and inflammatory or monocyte-derived cDCs or moDCs (Ly6C/G^+^) Neutrophils are CD3^−^CD19^−^CD11c^−^MHCII^−^Ly6C/G^+^ leukocytes. Treg cells were identified as CD3^+^CD4^+^Foxp3^+^CD25^+^ cells and percentage of cytokine positive cells was analyzed within the CD3^+^CD4^+^ population.

### Determination of statistical significance

4–5 mice were used for each condition studied in an individual experiment. Each substance was tested in at least 3 independent experiments. Animal numbers in the study were set up to reach a power of 97% with total BAL counts as endpoint when analyzing 6 animals per condition (calculation with G*Power® software 3.1.9.2). Data were analyzed after each experiment and also pooled. One-way ANOVA (analysis of variance) with Bonferroni’s multiple comparison test or Mann-Whitney t-test was performed with the GraphPad Prism® software. A *p* < 0.05 was considered to be significant.

### Data availability statement

The datasets generated during and/or analyzed during the current study are available from the corresponding author on reasonable request.

## Results

### Glucosteroids prove inefficient Th17 suppressors

**Figure 2:**
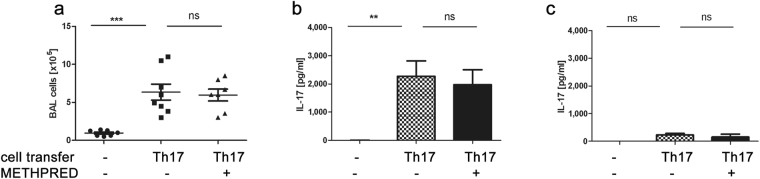
Methylprednisolone treatment fails to reduce Th17-dependent acute airway inflammation. Methylprednisolone (Methpred) treatment on d1 and 2 of the acute model fails to reduce (**a**) BAL cell count (absolute number ± SEM), antigen-specific IL-17A secretion (pg/ml + SEM, ELISA) of lung (**b**) or lymph node (**c**) cells. Pooled data of 3 independent experiments, total number of animals/group = 7–9. One-way ANOVA with the post-hoc Bonferroni’s multiple comparison test, **p < 0.01.

As inhaled and oral corticoids play a major role in bronchial asthma treatment, we initially tested methylprednisolone’s efficacy in suppressing acute Th17-mediated airway inflammation in our mouse models of acute and chronic Th17-dependent airway inflammation (Fig. 1a). Within these models, the acute model is dependent on the transfer of *in vitro* polarized Th17 cells while in the chronic model, endogenous IL-17-producing cells are generated as witnessed by appearance of ROR-γt positive cells as early as day 2 after transfer (Fig. 1b). Parenteral corticoid application on d1 and d2 failed to decrease total BAL cellularity (confidence interval (CI) = 3.9–11.4 × 10^5^ vs. 3.8–10.4 × 10^5^, p > 0,05) (Fig. 2a), or IL-17A secretion in the lung (Fig. 2b) or lymph nodes (Fig. 2c) at d4, demonstrating steroid-resistance of Th17-induced airway inflammation in our acute model. IL-17A secretion in the lymph nodes was overall negligible with no significant differences noted between treatment groups (Fig. 2c). Methylprednisolone by similar route and application efficiently inhibited airway inflammation and antigen-specific IL-4 and IL-13 secretion from lymph node lymphocytes in a Th2 polarized airway inflammation model induced by treatment with HDM (see Supplemental Fig. 1a–c), demonstrating that the dose and application route of the methylprednisolone were sufficient to suppress a Th2-polarized asthmatic phenotype.

### Roflumilast, Azithromycin and AM80 fail to suppress Th17-dependent airway inflammation

**Figure 3:**
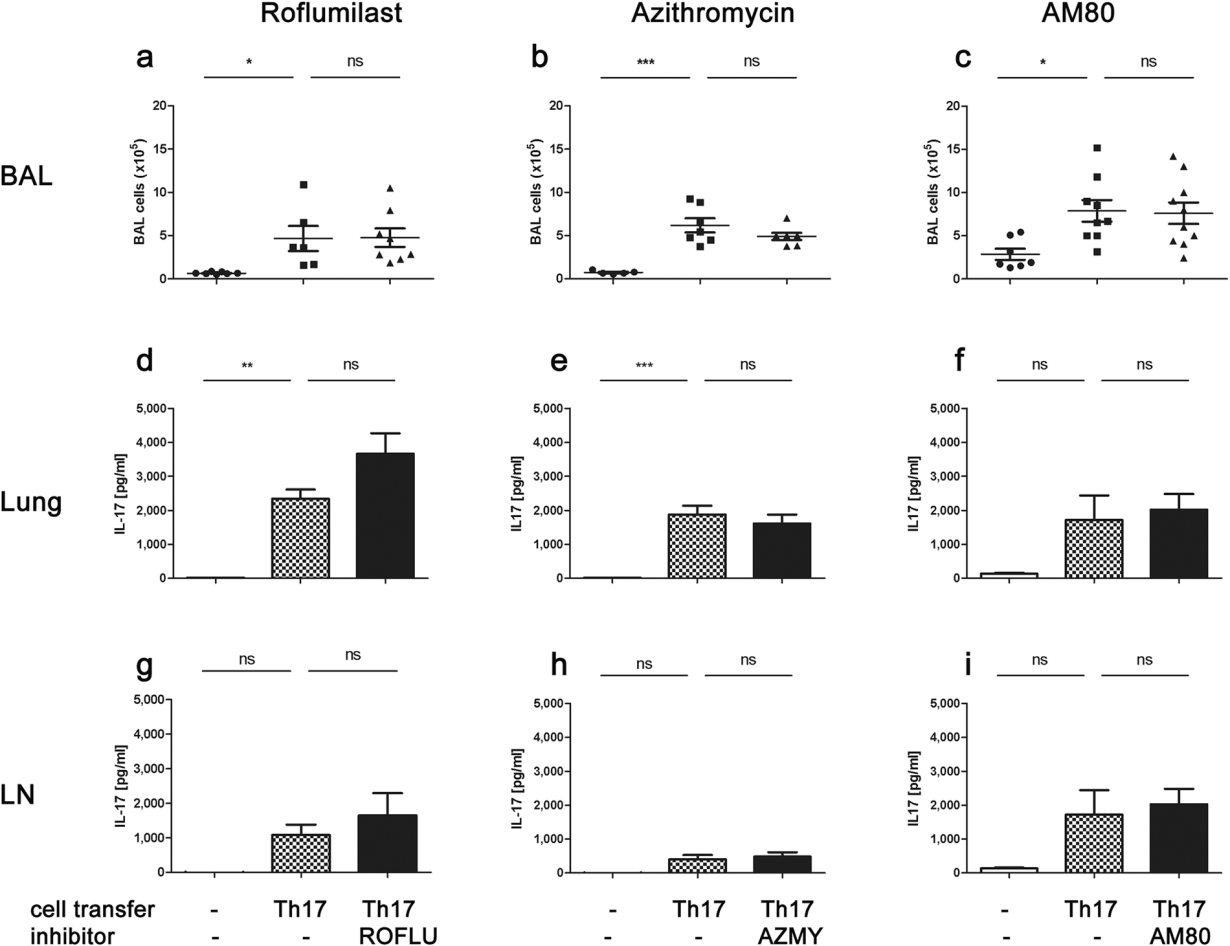
Roflumilast, azithromycin and AM80 fail to reduce Th17-dependent acute airway inflammation. Roflumilast (ROFLU), azithromycin (AZMY) and AM80 treatment on d1 and 2 (d-5, d-3, d0 and d3 for azithromycin) of the acute model fails to reduce BAL cell count (±SEM) (**a**–**c**), Ag-specific IL-17A secretion (pg/ml + SEM, ELISA) of lung (**d**–**f**) or lymph node lymphocytes **(g**–**i**). Pooled data from 2 independent experiments with a total of 7–10 animals/group. One-way ANOVA with the post-hoc Bonferroni’s multiple comparison test, *p < 0.05, **p < 0.01, ***p < 0.001.

Roflumilast and azithromycin are widely used anti-inflammatory agents in different inflammatory lung diseases (azithromycin) and COPD (roflumilast). AM80 is licensed in oncological diseases but showed efficacy in pre-clinical studies in the treatment of extra-pulmonary and pulmonary Th17-associated diseases. However, in our murine model of acute Th17-mediated airway inflammation which strictly depends on Th17 cell activation, we failed to note suppression of IL-17A-dependent airway inflammation by either substance (azithromycin: CI: 4.17–8.18 × 10^5^ vs. 3.9–5.89 × 10^5^; roflumilast: CI: 0.94–8.3 × 10^5^ vs. 2.21–7.31 × 10^5^; AM80: CI:4.97–10.76 × 10^5^ vs. 4.77–10.42 × 10^5^; Fig. 3a–c). Similarly, antigen-specific IL-17A secretion from lung (Fig. 3d–f) or lymph node (Fig. 3g–i) lymphocytes was unaffected by these substances. Interestingly, roflumilast (similar to methylprednisolone) successfully limited Th2-associated airway inflammation and antigen-specific secretion of IL-4 and -13 from lymph node lymphocytes (see Supplementary Fig. S1d–f), suggesting the failure to suppress airway inflammation was restricted to Th17-dependent airway inflammation for both substances.

### Rapamycin suppresses Th17-mediated airway inflammation in an acute model of Th17-dependent airway inflammation

**Figure 4:**
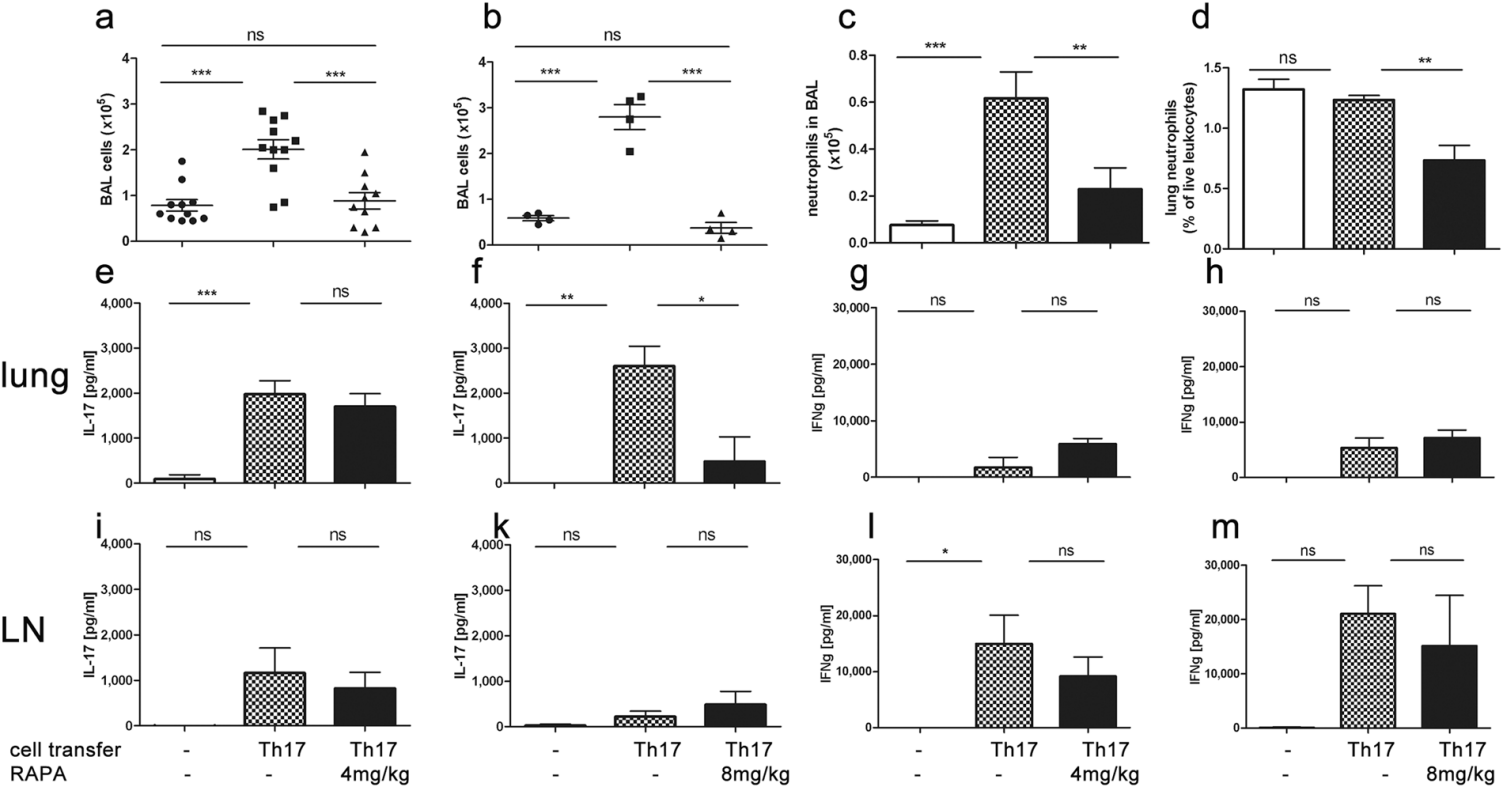
Rapamycin reduces Th17-dependent acute airway inflammation dose-dependently. Rapamycin treatment on d1 and 2 of the acute model resulted in a reduction of (**a**,**b**) BAL cell count (±SEM). (**c**) BAL neutrophils (±SEM) (asessessed by microscopic analyses of BAL cytospins) and (**d**) lung neutrophils (±SEM) (assessed by flow cytometry). Assessment of antigen specific cytokine secretion from lung (**e**–**h**) and lymph node cells revealed a dose-dependent reduction of IL-17A secretion in lung (**e**,**f**, ELISA) but not IFNγ (**g**,**h**, ELISA). Negligible amounts of IL-17A secretion were observed from lymph node cells (**I**,**k**, ELISA) with significant amounts of IFNγ secretion in this compartment (**l**,**m**, ELISA) but neither was regulated by rapamycin treatment. Pooled data of 1 (**b**,**d**,**f**,**h**,**k**,**m**) to 3 (**a**,**c**,**e**,**g**,**i**,**l**) independent experiments with a total number of 4–12 animals/group. One-way ANOVA with the post-hoc Bonferroni’s multiple comparison test, *p < 0.05, **p < 0.01, ***p < 0.001.

Intraperitoneal treatment with rapamycin on d1 and 2 of our treatment protocol (Fig. 1a) resulted in a significant decrease in the total number of BAL cells irrespective of the dosage (4 mg/kg vs. 8 mg/kg) (4 mg/kg untreated vs. treated CI: 1.54–2.48 × 10^5^ vs. 0.48–1.29 × 10^5^ Fig. 4a; 8 mg/kg untreated vs. treated CI: 1.93–3.67 × 10^5^ vs. 0.01–0.75 × 10^5^ Fig. 4b). Moreover, the number of BAL neutrophils as per enumeration of cytospins (4 mg/kg Fig. 4c) as well as percentages, as detected by flow cytometric analysis (8 mg/kg Fig. 4d) were decreased in the treated group (cytospins CI: 0.6–1.2 × 10^5^ vs. 0,06–0,29 × 10^5^; flow cytometry: CI: 1.12–1.33% of live leukocytes vs. 0.38–1% of live leukocytes). Rapamycin treatment did not affect the number of monocytes in the BAL fluid, but we observed a significant decrease in lymphocytes (CI: 0.08788 to 0.6262 × 10^5^) as well as eosinophils (CI: 0.001757 to 0.1422 × 10^5^) compared to vehicle treated groups (4 mg/kg, see Supplementary Fig. S2). Mucus production determined by means of PAS staining was not affected by rapamycin treatment (8 mg/kg, see Supplementary Fig. S3).

We observed a dose-dependent effect of rapamycin on IL-17A secretion, as rapamycin treatment with 4 mg/kg failed to significantly reduce IL-17A production in the lung (Fig. 4e), whereas treatment with 8 mg/kg led to a significant reduction in IL-17A secretion in the lung (Fig. 4f). In the lymph nodes we only detected negligible IL-17A quantities for all groups and no significant inter-group differences (Fig. 4i–k). IFNγ production in the lung showed insignificant amounts compared to LNs where significant secretion was detected (Fig. 4g–h). These results suggest that IL-17A and IFNγ secretions are spatially separated in our model. While IL-17A secretion occurs mainly in the lung (Fig. 4e–f), IFNγ secretion takes place mainly in the lymph nodes (Fig. 4l–m). We did observe a trend towards reduced IFNγ secretion in the LNs with both rapamycin doses, however the values failed to reach statistical significance (4 mg/kg CI: 3693–26295 pg/ml vs. 1465–16855 pg/ml; 8 mg/kg CI: 4620–37460 pg/ml vs. −14493–44747 pg/ml, Fig. 4l–m).

### Effect of rapamycin treatment on Th subpopulations

**Figure 5:**
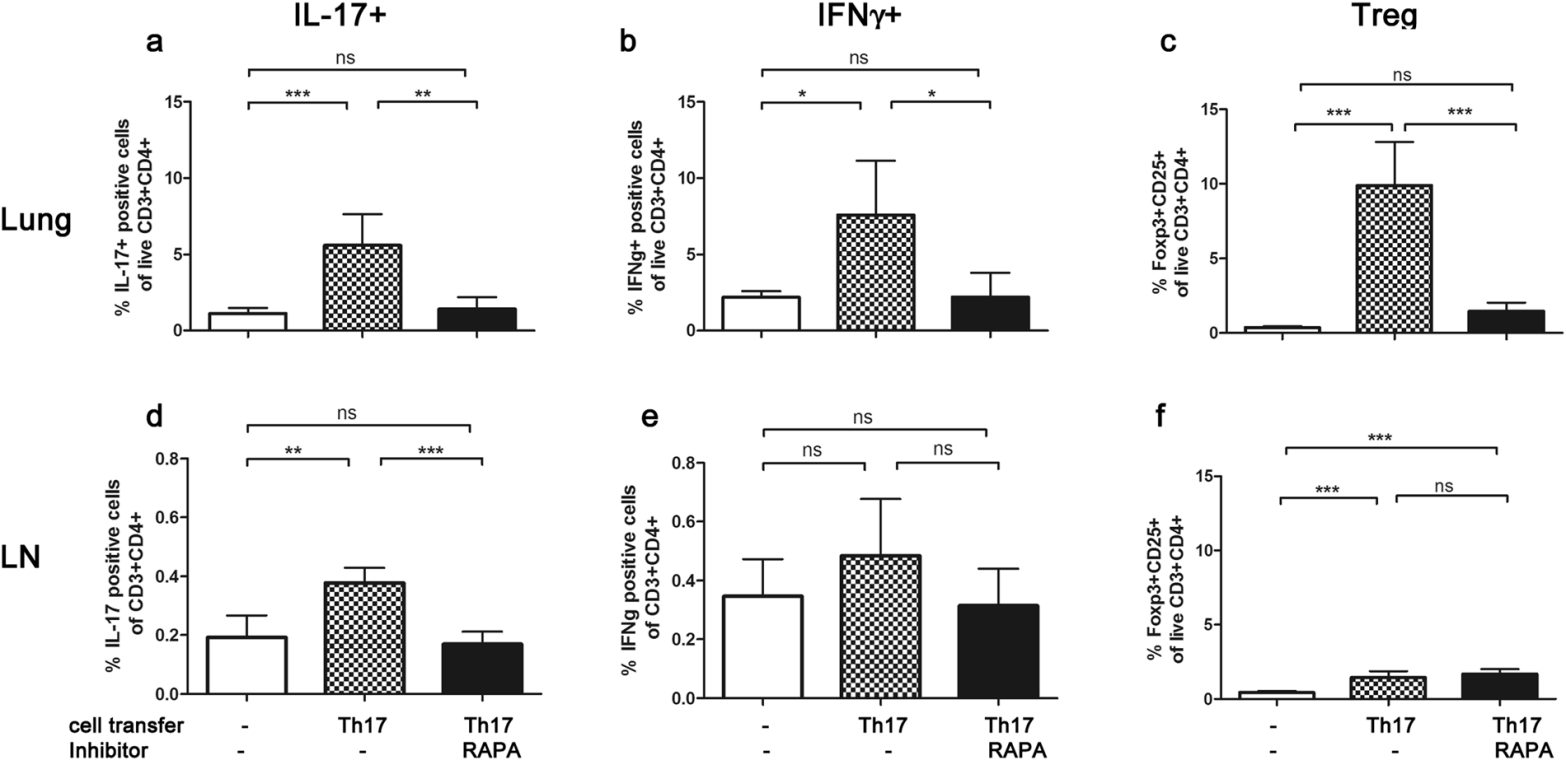
Rapamycin treatment decreases IL-17A^+^, IFNγ^+^ and regulatory T cells: Amount (% of T cells + SD) of IL-17A-producing (**a**,**d**), IFNγ-producing (**b**,**e**) and Tregs (**c**,**f**) were determined by means of flow cytometry in lung and LN from mice that underwent the acute model (RAPA, 8 mg/kg, d4). Data from one experiment with 5 animals per group. One-way ANOVA with the post-hoc Bonferroni’s multiple comparison test, *p < 0.05, **p < 0.01, ***p < 0.001.

The reduction of IL-17A and IFNγ by rapamycin was further supported by flow cytometric analyses of intracellular cytokine content of pulmonary and LN Th cells. Both Th17 (CD3^+^CD4^+^IL-17^+^ (Fig. 1d)) and Th1 (CD3^+^CD4^+^IFNg^+^ (Fig. 1d)) cell frequencies were significantly reduced by rapamycin in the lung (Fig. 5a,b). In the LNs Th17 cells were also significantly suppressed by rapamycin treatment with a strong trend towards reduction of Th1 cells (Fig. 5d,e). However, these concomitant changes of Th1 and Th17 cells argue against a change in Th1/Th17 balance to underlie the effect of rapamycin treatment.

The balance of regulatory T cells (Treg) and Th17 cells is tightly linked and rapamycin is described to alter this balance^24,25^. Therefore, we additionally determined the effect of rapamycin treatment (8 mg/kg) on Tregs in lung and LNs from mice that underwent the acute protocol by means of flow cytometry (Fig. 5). Interestingly, Treg frequencies (CD4^+^CD3^+^CD25^+^Foxp3^+^ (Fig. 1d)) in the lung were decreased by rapamycin treatment, similar to our observations for Th17 and Th1 cells (Fig. 5c). In the LNs, Tregs were unaffected by rapamycin treatment, however, their overall numbers were small and animal numbers might have been underpowered to detect significant differences (Fig. 5f).

### Effects of rapamycin treatment on DC subpopulations

To characterize the effect of rapamycin on antigen-presenting cells, we performed flow cytometric analyses of dendritic cell (DC) subpopulations (pDCs, cDCs, moDCs) in lung parenchyma. Among live, CD3-CD19- cells pDCs were identified as CD11c^low^B220^+^LyC/G^+^, and cDCs as MHCII^high^CD11c^high^. cDCs were further subdivided in steady-state cDCs (Ly6C/G^−^) and moDCs (Ly6C/G^+^) (Fig. 1c).

**Figure 6:**
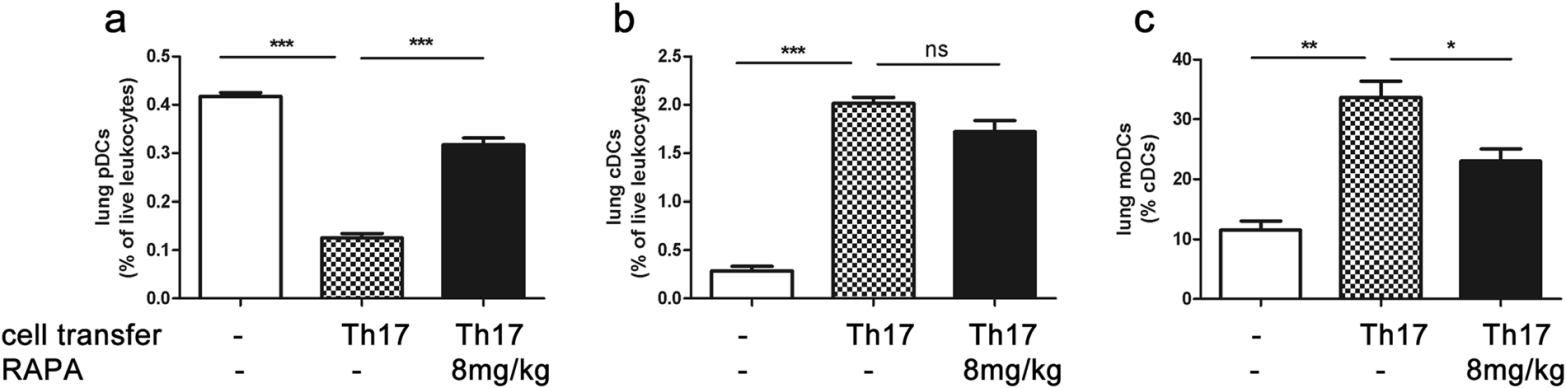
Dendritic cells’ dynamic upon Rapamycin treatment: pDCs (% + SEM) return to numbers comparable to the negative control group (**a**) and, while the total cDC numbers remain constant (**b**), there is a significant decrease in the percentage of moDCs (% + SEM) (**c**). Data from one experiment with 4 animals/group. One-way ANOVA with the post-hoc Bonferroni’s multiple comparison test, *p < 0.05, **p < 0.01, ***p < 0.001.

Rapamycin treatment increased the percentage of pDCs to values comparable to the naïve controls (Fig. 6a). While this does not hold true for total cDCs (Fig. 6b), within the cDC population the proportion of moDCs decreases significantly upon rapamycin application (Fig. 6c).

### Rapamycin treatment reduces CXCL-1/KC levels but fails to reduce other cytokines involved in Th17 differentiation or downstream effects of IL-17A

**Figure 7:**
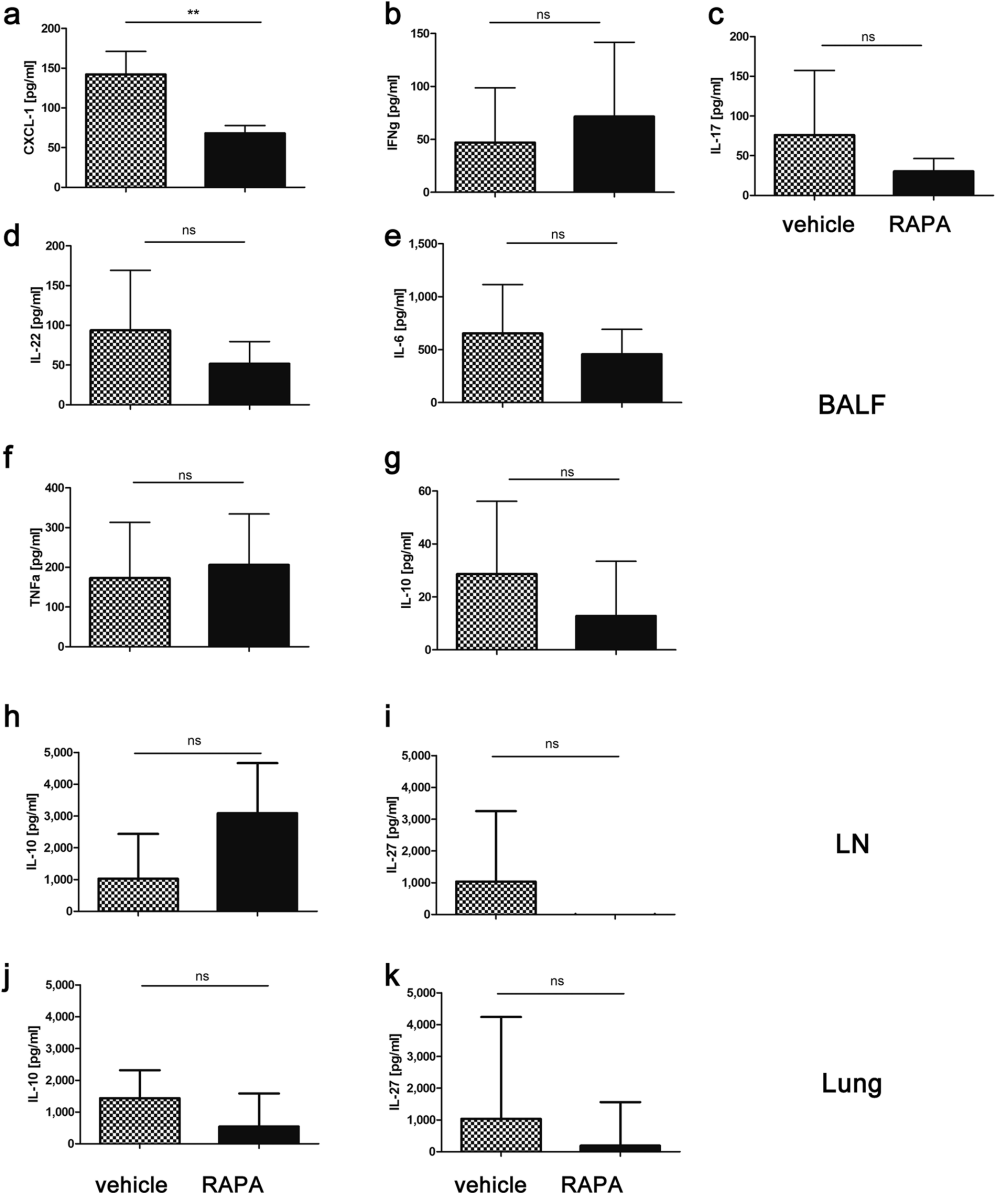
Influence of Rapamycin on Th17/IL-17A-related cytokines and chemokines in BALF, lung and LN: Mediator concentrations (pg/ml + SD) were measured in BALF (**a**–**g**) and supernatants of OVA-specific stimulated LN (**h**,**i**) or lung (**j**,**k**) cells from mice that underwent the acute model (RAPA 8 mg/kg, d4) by means of cytometric beads array (**b**–**k**) or ELISA (**a**). Data from one experiment with 5 animals per group. Mann-Whitney t-test, **p < 0.01.

For mechanistic insight into mediators responsible for the effects we observed for rapamycin treatment, we chose to analyze cytokines in BALF or after antigen specific stimulation of lung and lymph node cells, known to be involved in Th17 differentiation and/or downstream effects of IL-17A. In accordance with the reduction of neutrophil influx into the BALF by rapamycin treatment, we found a significant reduction of CXCL-1, which is an important neutrophil recruitment factor, in BALF (Fig. 7a).

In contrast IFNg-producing T-cells in lung (Fig. 5b), we found that BALF IFNγ content was not changed (Fig. 7b). Whilst there were trends for reductions of IL-17A and IL-22 content of BALF by rapamycin, these changes failed to reach statistical significance (Fig. 7c,d). Discrepancy between flow cytometry and ELISA data could be due to IL-17A and IFNg production by other cells than Th cells, which we did not examine in flow cytometry. Regardless of the source of the cytokines, since both IL-17A and IFNg seem to be regulated in the same manner by rapamycin in the different materials tested, our results argue against a strong shift in the balance of Th1- vs Th17-associated cytokines by rapamycin treatment.

For some cytokines, rapamycin treatment did not confer any changes: neither BALF IL-6 nor TNFα, which can directly or via IL-23 promote Th17 development and thus IL-17A secretion, were affected by rapamycin treatment (Fig. 7e,f). Similarly, neither BALF nor tissue IL-10 (Fig. 7g,h,j), or tissue IL-27 levels (Fig. 7i,k), known to antagonize Th17 development, were altered by rapamycin treatment.

Finally, IL-21, known to also favor Th17 differentiation, but also IL-4 and IL-5, were below detection limits in our model in BALF, rendering conclusions with regards to an altered Th2/Th17 balance by rapamycin impossible (data not shown).

### Rapamycin fails to significantly reduce Th17-dependent chronic airway inflammation

**Figure 8:**
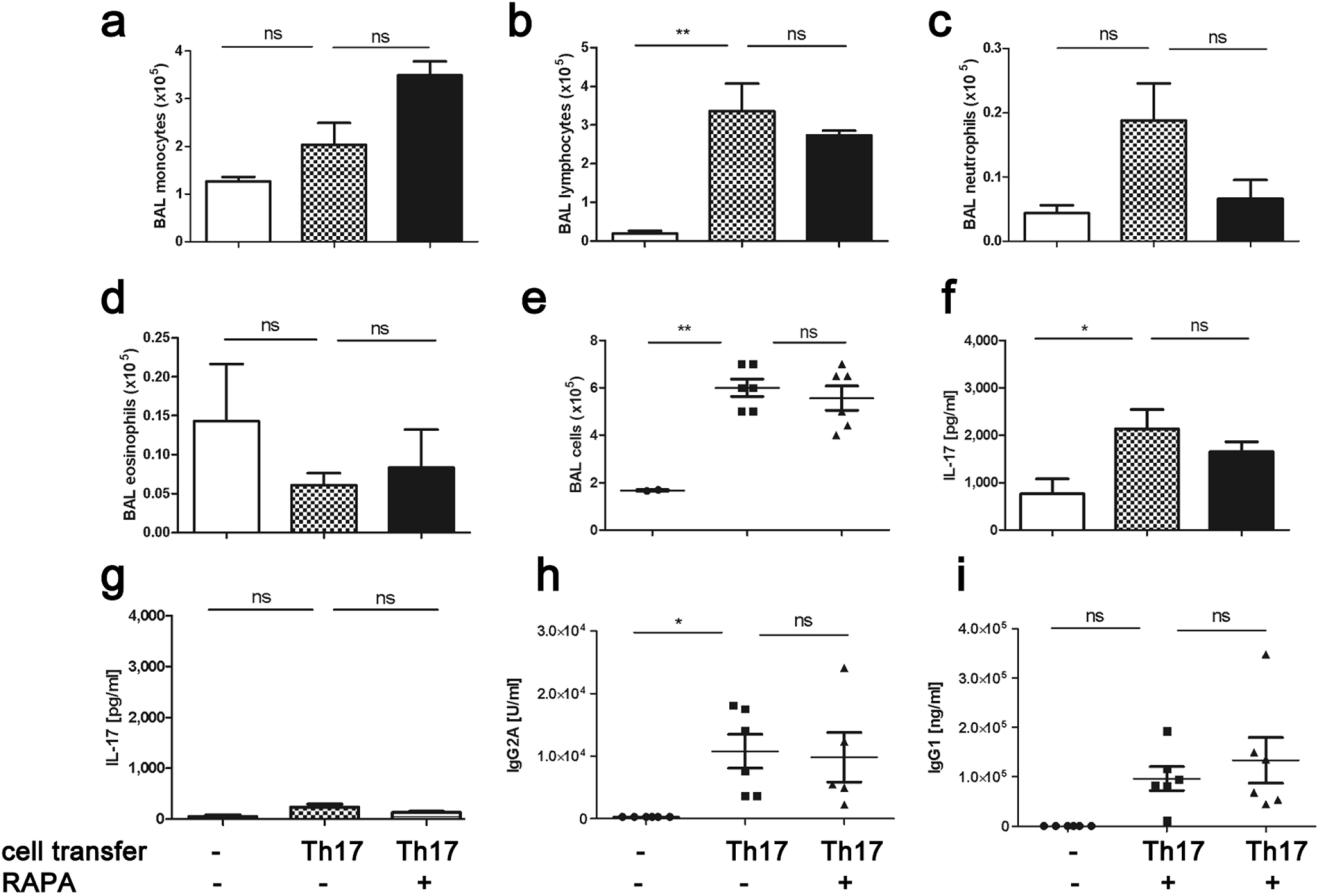
Rapamycin fails to reduce Th17-dependent chronic airway inflammation. In the chronic inflammation model rapamycin treatment did not impinge either total BAL cellularity (**a**–**e**), Ag-specific IL-17A production (pg/ml + SEM, ELISA) in the lung (**f**), LN (**g**) or Ag-specific IgG2a (U/ml) and IgG1 (ng/ml) production (**h**,**i**). Data from one representative experiment with a total of 5–6 animals/group. One-way ANOVA with the post-hoc Bonferroni’s multiple comparison test, *p < 0.05, **p < 0.01, ***p < 0.001.

Given our promising results in the acute model of Th17-dependent airway inflammation, we assessed the effects of rapamycin in a chronic airway inflammation model.Here the pulmonary inflammation after the second challenge (d18/d19) relies on endogenously generated IL-17-producing cells due to the first challenge (d2-3), as we could demonstrate using RORgt-reporter mice^16^ as acceptors (Fig. 1a,b). As opposed to the acute model, rapamycin was unable to reduce Th17-dependent chronic airway inflammation (Fig. 8). Despite an increase in the number of monocytes in BAL (Fig. 8a) and accompanying reductions in lymphocytes (Fig. 8b) and neutrophils (Fig. 8c), these effects were not significant. Although they do not constitute an important feature of this model, we also assessed eosinophil numbers, which were not affected (Fig. 8d). As a result we did not see a significant change in total cell numbers upon treatment (Fig. 8e). Similar to our observations in the acute model, IL-17A secretion was mainly detected in the lung (Fig. 8f) and negligible in the lymph nodes (Fig. 8g). While there was a trend towards reduced IL-17A secretion by lung lymphocytes, it failed to reach statistical significance (Fig. 8f). Lymph node secretion was not altered significantly either (Fig. 8g). Accordingly, rapamycin treatment was also not able to reduce the accompanying antigen-specific immunoglobulin secretion (Fig. 8h and i).

## Discussion

Our data show a strong anti-inflammatory efficacy of rapamycin in treating acute Th17-dependent airway inflammation reducing neutrophils, eosinophils and lymphocytes in BALF. This anti-inflammatory effect is accompanied by suppression of Th1, IL-17A, Th17 and Tregs as well as moDcs and an increase in pDCs. Mechanistically, rapamycin treatment in our acute model was accompanied by reduction of BALF CXCL-1/KC levels suggesting an effect on the IL-17A-IL-8 axis. Yet, we failed to demonstrate a significant effect of rapamycin on chronic Th17-dependent airway inflammation. Furthermore, we failed to prove efficacy of roflumilast, azithromycin and AM80 on acute Th17-dependent airway inflammation.

IL-17A mediates strong chemo-attractive effects on neutrophils via induction of IL-8 secretion from different stromal cells^26^. These events are visible in our data on the effects of rapamycin on airway neutrophilia (Fig. 4c,d). IL-17A-induced IL-8 release has been shown to be refractory to steroid treatment^27^ which is mirrored by our own results on the effects of methylprednisolone on BAL cell counts and IL-17A secretion (Fig. 2). By assessment of CXCL-1/KC – the mouse homologue to IL-8 - in BAL fluid we could demonstrate that in our model rapamycin, via reduction of IL-17A, reduces epithelial IL-8 secretion (Fig. 7a) thereby leading to reduced neutrophilia, an effect not exerted by methylprednisolone. A similar connection of rapamycin to the IL-17A-IL-8 axis in airway inflammation is not surprising, given rapamycin’s known suppression of IL-17A in different model systems^28–30^. Yet – to our knowledge – it hasn’t been demonstrated in a Th17-driven pulmonary inflammation model. We were not able to identify changes in several other mediators we analyzed. At this stage, we are thus unable to connect the effects of rapamycin on Th cells and DCs to specific changes in cytokines, as we have been able to show for neutrophils via the IL-17A-KC/IL-8 axis.

Recent literature provided strong evidence that mTOR, rapamycin’s target, takes both metabolic and cytokine cues from the environment, thereby tipping the immune balance towards effector cells’ differentiation^31–33^. Some of these findings could provide explanations for the results we observed: Different publications demonstrate a dependency of Th17 development on mTOR signaling^34^, mTOR complex 1 (mTORC1) and the small GTPase Rheb^13^. mTOR signaling has been shown to affect cytokine secretion by Th17 cells^35^ and their proliferation^36^, suggesting that these effects might underlie our findings. In that line, we did observe significantly reduced IL-17A secretion by pulmonary lymphocytes (Fig. 4), however, we did not formally address changes in proliferation of the transferred Th17 cells induced by treatment with rapamycin in our model system.

Different publications have demonstrated a role for mTOR signaling in the balance between Tregs and Th17 cells *in vitro* and *in vivo* ^37–39^. In that line, we showed that the pDC population becomes partly restored after rapamycin treatment (Fig. 6a). Lung pDCs have been shown to support the generation of regulatory T cells and transfer of pDCs prevents development of airway hyperreactivity and inhibits the effector phenotype *in vitro* in murine asthma models^40^. However, the increase in pDCs in our model was not accompanied by an increase of Tregs in lung or LNs. To the contrary: we observed a reduction of lung Tregs while LN Tregs were unaffected. Thus, the balance of Th17/Tregs via mTOR signaling does not seem to underlie the anti-inflammatory effect of rapamycin in our model system.

Several aspects might contribute to rapamycin’s lack of efficacy in our chronic model: Some of these aspects pertain to the experimental approaches we took. Firstly, we have to acknowledge that we set up our study with animal numbers that would give us a power of 97% using BAL total cell counts as endpoint. Sub-analyses showed that neutrophil counts showed a strong tendency towards reduced numbers in our chronic model (Fig. 8c) and there was a trend towards reduced lymphocytes (Fig. 8b), accompanied by a trend towards reduced antigen-specific IL-17A in the lung (Fig. 8f), similar to the effects we observed in the acute model where these effects had reached statistical significance (Fig. 4c and f, Supplemental Fig. S2). However to reach a power of 95% with neutrophils as endpoint, we would have had to double animal numbers which we considered not feasible when planning the experiments due to animal welfare constraints.

Another rather simplistic explanation pertains to timing and dosage: in our model we have shown that priming of naive, endogenous T cells towards a Th17 phenotype takes place (ref.^6^ and Fig. 1). Our own data demonstrate an early differentiation of endogenous Th17 cells, starting on d2-4 of our model (Fig. 1). However, this does not exclude that the time of rapamycin treatment we employed might not be sufficient to control terminal Th17 differentiation and thus leave the ensuing chronic inflammation unaltered.

Different groups have demonstrated a dose-dependent effect of rapamycin, depending on the dose regimen, rapamycin capable of inducing a regulatory phenotype or contributing to the development of central memory/effector memory T lymphocytes^41–43^. The dose-dependent effects of rapamycin treatment we observed within our acute model are in line with those findings and render it possible that another dosing regimen might increase efficacy of rapamycin in the chronic model as well.

Additionally, there are mechanistic aspects to be considered when querying the reason why rapamycin fails to efficiently suppress chronic airway inflammation in our model. While acute Th17-dependent airway inflammation is completely dependent upon transfer and activation of polarized Th17 cells, the ensuing chronic airway inflammation could rely on additional factors besides the development of endogenous Th17 cells; factors that could be rapamycin resistant. A combination approach of rapamycin together with a second immunomodulatory drug could enhance its suppressive capacity. In that line, Kim *et al*. used a combination of rapamycin and all-trans-retinoic acid to re-differentiate memory Th2 cells towards a Treg phenotype and thereby reduce allergic airway inflammation^14^. Kim’s data suggest that a combination of rapamycin and AM80 might improve efficacy of retinoic-acid receptor agonism on Treg generation^14^, an interesting approach for our chronic model where rapamycin by itself lacked efficacy (Fig. 8).

In another line of thought, Amiel *et al*. were able to demonstrate that mTOR inhibition extends the activated DCs’ lifespan, enhancing the time frame in which they exhibit an activated phenotype^44,45^. Adding another layer of complexity, mTOR is the core member of two protein complexes: mTORC1 and mTORC2. Recent data provide evidence that mTORC2 deficient myeloid DCs have an enhanced Th1 and Th17 stimulatory activity^46,47^ Recently, Sinclair *et al*. describe distinct effects of mTOR specifically in lung DCs and alveolar macrophages: using CD11c-specific mTOR knockout mice they showed that mTOR plays an important role in homeostasis and subset composition of antigen-presenting cells in the lung by controlling their survival in a translation-independent manner via mTORC1 at steady-state. During inflammation in a Th2-driven model cell-specific depletion of mTOR led to Th17-derived pulmonary inflammation and neutrophilic influx^48^. It seems conceivable that the effects of rapamycin in our acute inflammation model are due to a direct inhibition at the T cell level which is supported by our flow cytometric data where a broad suppressive effect of rapamycin is observed, affecting not only Th17 cells but also Th1 cells and Tregs. Possibly, the lack of effect in the chronic inflammation model could be explained by enhanced activity of DCs via rapamycin-dependent prolongation of survival which would support extended Th17 priming. Whether this effect is mediated through mTORC1 or mTORC2 in an already established Th17 response would need to be explored While we did assess the DC populations’ dynamic upon mTOR inhibition with rapamycin, assessments of their lifespan are yet to be performed.

Our results demonstrate clearly the difficulties in extrapolating data from one model system to another. Despite the fact that pre-existing data suggested efficacy for all tested substances in a Th17-dependent system, roflumilast, methylprednisolone, azithromycin and AM80 did not suppress Th17-dependent airway inflammation in our acute model: They did not only fail to limit total cell influx in BAL fluid, but had no significant effect on the number of each cell type (monocytes, lymphocytes, neutrophils, eosinophils) alone, despite being anti-inflammatory drugs (see Supplementary Fig. S4). We could show that methylprednisolone and roflumilast are very efficacious in Th2-driven lung disease model (see Supplementary Fig. S1). Efficacy of these agents against airway inflammation in asthma, but presumable also COPD could rely on this effect - amongst other known anti-inflammatory effects for these agents which have been described before. The lack of efficacy of these agents against a Th17-dependent inflammation suggests that the treatment failure subgroups of patients with asthma or COPD experience when treated with these anti-inflammatory drugs is due to lack of Th17-specific anti-inflammatory effects of these agents. Our findings thus underline the need for more specific treatment options and corroborate their the importance regarding rapamycins’ Th17 specific actions.

The effects of AM80 were described to be inhibitory on Th17 development by regulating transcription factors^49^ and showed efficacy in models of graft-versus-host-disease and experimental autoimmune encephalitis^12,50^. In that context, we might be faced with a relative lack of inhibitory activity: while a full-blown Th17-mediated inflammatory response induced by transferring already differentiated T cells might be too strong to be suppressed, whereas an incipient response might still be susceptible to modulation. Thus, testing the substances which failed to show efficacy in our acute Th17-dependent model system in other model systems with a less pronounced but measurable Th17-dependent phenotype might serve to further assess their possible modulation of Th17-dependent airway inflammation.

Furthermore, some of the data suggesting an anti-Th17-effect were assessed in simple model systems which might not transfer to a complex model system such as our model. For instance, considerable previous data on azithromycin were collected in *in vitro* assays using IL17-stimulated human airway smooth muscle cells^8^. The effects to be expected by roflumilast treatment in our model system were unclear, since on one hand it is described to be anti-inflammatory inhibiting T cell proliferation and epithelial IL-6 secretion^51,52^, but on the other hand cAMP, elevated by phosphodiesterase inhibitors like roflumilast, plays an important role in driving Th17 differentiation^53^. In our hands Roflumilast did not inhibit acute Th17-driven airway inflammation and a recent publication examining dendritic cells in the context of roflumilast treatment even report a supporting role for this drug in Th17 development^54^, corroborating our data.

Our results demonstrate that Th17-dependent airway inflammation is difficult to treat. This might explain the clinically notoriously difficult disease course of those pulmonary diseases associated with Th17-polarization. These pathologies such as severe asthma, lung transplant rejection, transplant-associated as well as transplant-independent bronchiolitis obliterans and cystic fibrosis significantly contribute to overall pulmonary disease burden. Moreover, treatment options are limited and often, as in the case of steroids, unsuccessful even in reducing the speed of disease progression, let alone halting its course. Despite our failure to demonstrate statistically significant effects of rapamycin treatment on chronic airway inflammation, which we regard as a prerequisite for further pre-clinical testing, we believe that our results on rapamycin’s effects on acute Th17-dependent airway inflammation and the trends, we observed in the chronic model constitute a promising starting point for further pre-clinical testing. Although rapamycin treatment in our model failed to affect all aspects of pulmonary inflammation (like mucus production) we did observe strong effects on different immune cells and cytokine production. Several experimental studies describe the efficacy of rapamycin to suppress inflammatory processes via different mechanisms in the lung in the context of other disease entities like allergic airway inflammation, acute lung injury and cystic fibrosis^29,55,56^, adding evidence to our conclusion regarding rapamycin’s usefulness in treating inflammatory lung diseases. Combination therapies as well as different treatment protocols, as pointed out above, might enhance rapamycin’s efficacy in the treatment of chronic airway inflammation and could thus pave the way for a novel treatment option for pulmonary diseases for which currently treatment options are scarce.

## Electronic supplementary material

Supplementary Dataset
