# Supplementary material for: Suppression of Th17-polarized airway inflammation by rapamycin
Source: Sci Rep. 2017 Nov 10;7:15336. doi: 10.1038/s41598-017-15750-6 (PMC5681547; doi:10.1038/s41598-017-15750-6)
Supplement: Supplementary file 1 — Supplementary Dataset [file 41598_2017_15750_MOESM1_ESM.doc]

Oana Joean, Anja Hueber, Felix Feller, Adan Chari Jirmo, Matthias Lochner, Anna-Maria Dittrich, Melanie Albrecht

| **Target** | **Clone** | **Supplier** |
| --- | --- | --- |
| CD3 | 145-2C11 | BD Pharmingen |
| MHCII | 2G9 | BD Pharmingen |
| B220 | RA3-6B2 | BD Pharmingen |
| Ly6C/G | RB6-85C | BD Pharmingen |
| IL-17A | TC11-18H10.1 | BD Pharmingen |
| CD19 | 6D5 | BioLegend |
| CD45 | 30-F11 | BioLegend |
| CD11c | N418 | BioLegend |
| GFP | polyclonal rabbit | molecular probes |
| Rabbit-IgG | polyclonal goat | molecular probes |
| CD4 | RM4-5 | BioLegend |
| CD25 | 3C7 | eBioscience |
| IFNγ | XMG1.2 | BD Pharmingen |
| Foxp3 | FJK-16S | eBioscience |

**Supplementary Table S1: Antibodies used in flow cytometric analyses**

**
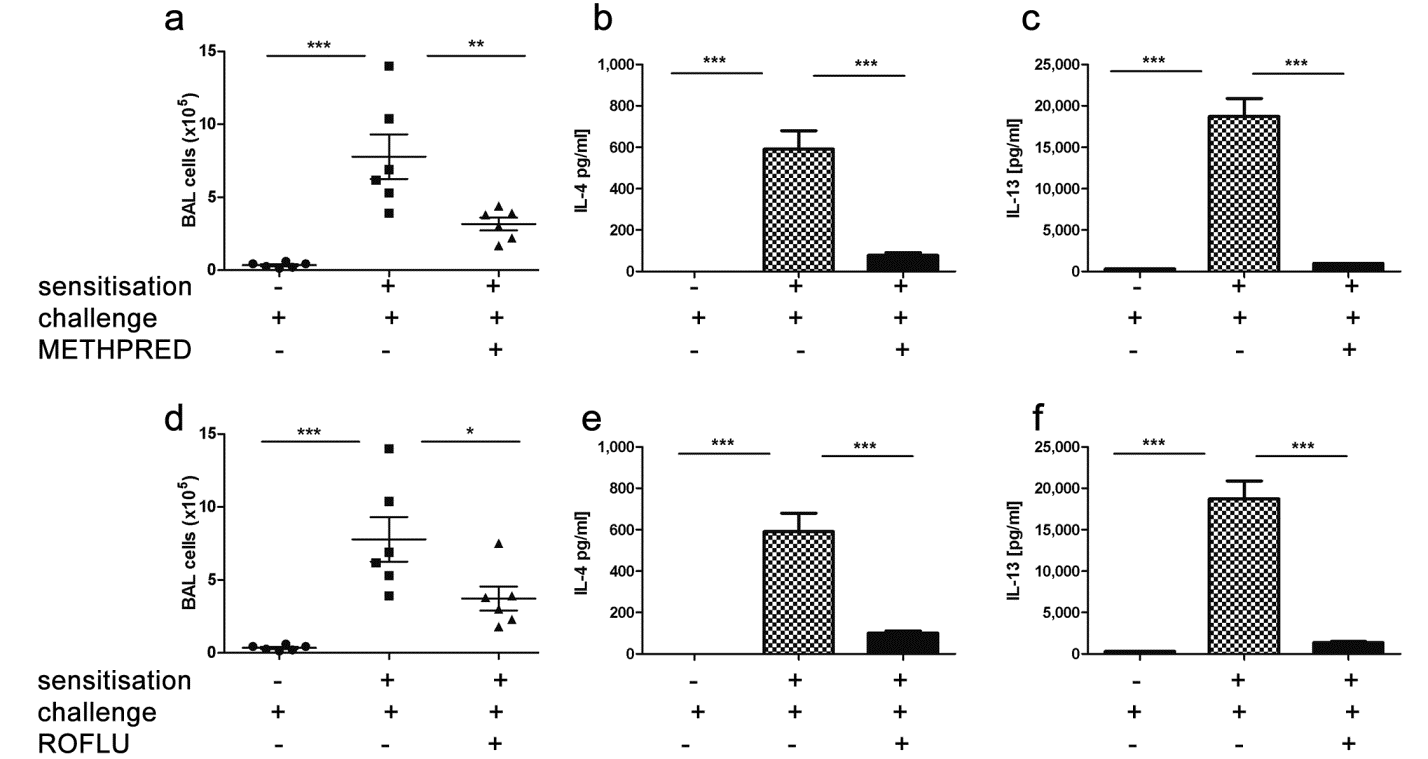
**

**Supplementary Figure S1: Methylprednisolone and Roflumilast strongly inhibit Th2-mediated acute airway inflammation.** Both methylpsrednisolone and roflumilast treatment in a model of acute Th2-driven lung inflammation reduce: BAL cell count (a,d), LN Ag-specific secretion of IL-4 (pg/ml + SEM) (b, e) and IL-13 (pg/ml + SEM) (c, f), respectively. Data from two representative experiments with 5 animals/group. One-way ANOVA with the post-hoc Bonferroni’s multiple comparison test, * p<0.05, **p<0.01, ***p<0.001


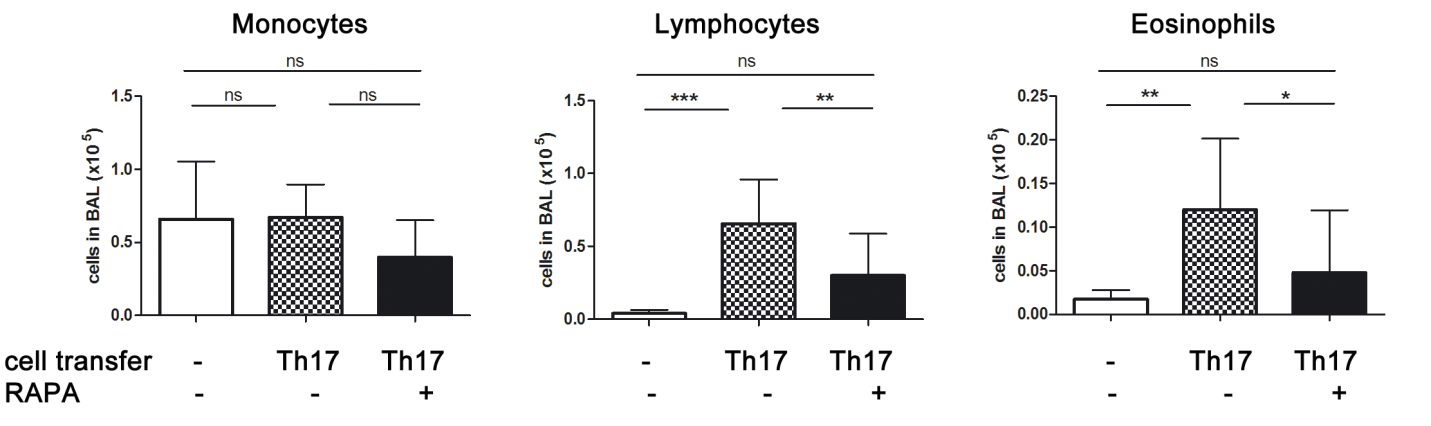


**Supplementary Figure S2: Rapamycin treatment affects lymphocyte and eosinophil counts in BALF in the acute model (RAPA 4mg/kg).** Pooled data of 3 experiments, n=10-11 per group. One-way ANOVA with the post-hoc Bonferroni’s multiple comparison test, * p<0.05, **p<0.01, ***p<0.001


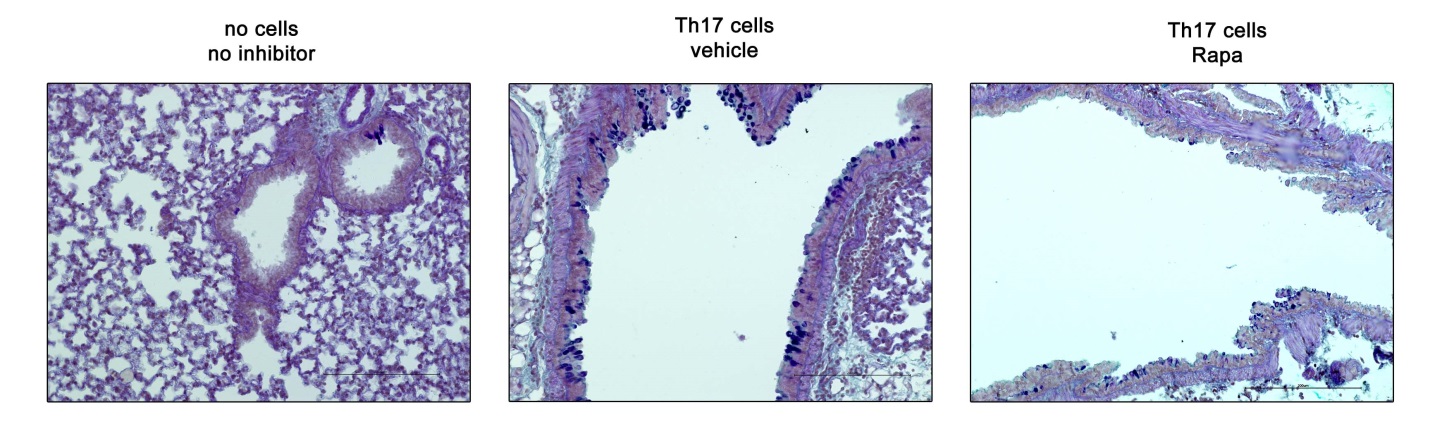


**Supplementary Figure S3: Mucus production and goblet cell hyperplasia is not affected by Rapamycin treatment in acute model**

Periodic Acid Schiff (PAS)-staining of lung sections from mice that underwent acute model (RAPA 8mg/kg). Magnification 20x, scale bar 200µm; Keyence microscope BZ9000


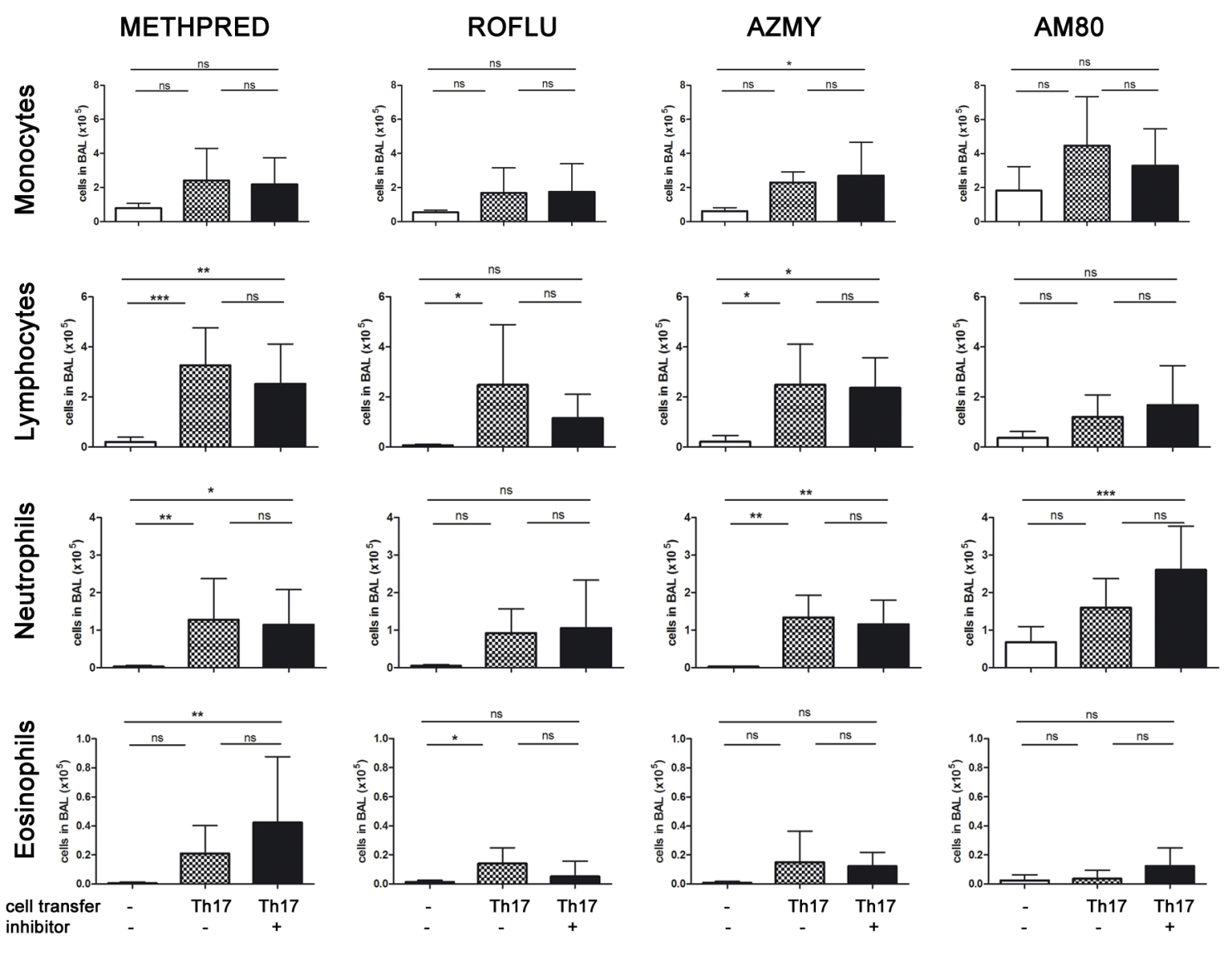


**Supplementary Figure S4: Methylprednisolone, Roflumilast, Azithromycin and AM80 fail to influence cell counts in BALF in acute model.**

Methylprednisolone (METHPRED), roflumilast (ROFLU), azithromycin (AZMY) and AM80 treatment on d1 and 2 (d-5, d-3, d0 and d3 for azithromycin) of the acute model fails to reduce differential BAL cell count (± SEM). Pooled data from 2-3 independent experiments with a total of 7-10 animals/group. One-way ANOVA with the post-hoc Bonferroni’s multiple comparison test, * p<0.05, ** p<0.01
